# Downregulation of TLR4 by miR-181a Provides Negative Feedback Regulation to Lipopolysaccharide-Induced Inflammation

**DOI:** 10.3389/fphar.2018.00142

**Published:** 2018-02-26

**Authors:** Kangfeng Jiang, Shuai Guo, Tao Zhang, Yaping Yang, Gan Zhao, Aftab Shaukat, Haichong Wu, Ganzhen Deng

**Affiliations:** Department of Clinical Veterinary Medicine, College of Veterinary Medicine, Huazhong Agricultural University, Wuhan, China

**Keywords:** acute lung injury, miR-181a, LPS, NF-κB, ROS

## Abstract

Acute lung injury (ALI) is a progressive clinical disease with a high mortality rate, and characterized by an excessive uncontrolled inflammatory response. MicroRNAs (miRNAs) play a critical role in various human inflammatory diseases, and have been recognized as important regulators of inflammation. However, the regulatory mechanisms mediated by miRNAs involved in Lipopolysaccharide (LPS)-induced inflammation in ALI remain hazy. In this study, we found that miR-181a expression in the lung tissues of ALI mice and LPS-stimulated RAW 264.7 macrophages is dramatically reduced. We also show that over-expression of miR-181a significantly decreased the production of inflammatory cytokines, such as IL-1β, IL-6, and TNF-α, whereas inhibition of miR-181a reversed this decrease. Moreover, miR-181a inhibits NF-κB activation and accumulation of reactive oxygen species (ROS) by targeting TLR4 expression. We further verify that miR-181a suppresses TLR4 expression by binding directly to the 3′-UTR of TLR4. Therefore, we provide the first evidence for the negative regulation of miR-181a in LPS-induced inflammation via the suppression of ROS generation and TLR4-NF-κB pathway.

## Introduction

Acute lung injury (ALI) is an excessive uncontrolled inflammatory response in lung tissues caused by various clinical disorders, including pneumonia, major trauma, and sepsis (Shen et al., 2009; Xiao et al., 2015; Yao et al., 2017). ALI is mainly characterized by the over-expression of inflammatory mediators and numerous neutrophils infiltration, which finally leads to pulmonary edema, hemorrhage, and even gas exchange impairment (Rubenfeld et al., 2005).

It is well established that inflammatory stimuli from microbial pathogens, such as endotoxin, are widely considered to be one of the causes of severe pneumonia (Rojas et al., 2005). Lipopolysaccharide (LPS), a potent endotoxin, is a major biologically active ingredient of the Gram-negative bacterial cell wall and plays a pivotal role in inflammatory responses (Dreyfuss and Ricard, 2005). Previous studies have demonstrated that LPS could induce ALI by activating TLR4/NF -κB signaling pathway,which regulates the transcription of pro-inflammatory cytokines, such as IL-1β, IL-6, and TNF-α (Wu et al., 2016).These cytokines can induce innate immune response and cause serious injury to the lung tissues, ultimately result in ALI (Herold et al., 2011; Gandhi and Vliagoftis, 2015).

MicroRNAs (miRNAs) are a class of evolutionarily conserved, single strand and small non-coding RNA molecules, approximately 20–25 nucleotides (nts), which mainly regulate gene expression in a post-transcriptional level (Chen and Rajewsky, 2007; Leuenberger et al., 2016). Once in their mature form, miRNAs interact specifically with the 3′-untranslated regions (3′-UTRs) of target mRNAs, resulting in silencing of their functions through mRNA degradation or translational inhibition (Griffiths-Jones et al., 2006; Xu et al., 2016). A number of miRNAs have been demonstrated to have a notable impact on the magnitude of the inflammatory response by targeting signal transduction proteins or directly targeting mRNAs that encode pro-inflammatory cytokines following pathogenic microbes infection (O’Connell et al., 2012). Moreover, some studies also suggested that some miRNAs are involved in the Toll-like receptor (TLR) pathway, and it is likely that they modulate signaling transduction during the inflammatory response (Nahid et al., 2011). Recently, it has been reported that miR-181a is associated with tumor growth and immune response (Seoudi et al., 2012), and suppresses TNF-α-induced transcription of pro-inflammatory genes (Zhao et al., 2012). miR-181a also negatively regulates immune responses in DCs (Zhu et al., 2017), and influences differentiation of T helper cell and activation of macrophages (Ghorbani et al., 2017). More importantly, miR-181a could function as an apoptosis promoter in the pathogenesis of ALI (Li et al., 2016). These findings prompted us to investigate the role of miR-181a in immune responses to LPS both *in vivo* and *in vitro*. Here, we found that LPS treatment highly decreased the expression of miR-181a in lung tissues and macrophages. We also showed that transfection with miR-181a mimics or inhibitors resulted in a down-regulation or up-regulation in pro-inflammatory cytokines, such as IL-1β, IL-6, and TNF-α. Further experiments demonstrated that miR-181a decreased TLR4 expression by binding directly to the 3′-UTR of TLR4. This study revealed that miR-181a could be an important negative regulator of inflammation and shed new light on therapeutic approaches toward some inflammatory diseases including ALI.

## Materials and Methods

### Reagents

Lipopolysaccharide (*Escherichia coli* 055:B5) was purchased from Sigma-Aldrich (St. Louis, MO, United States). Mouse TNF-α, IL-1β, and IL-6 enzyme-linked immunosorbent assay (ELISA) kits were purchased from ImmunoWay Biotechnology (Newark, DE, United States). The myeloperoxidase (MPO) determination kits were obtained from Nanjing Jiancheng Bioengineering Institute (Nanjing, China). Phospho-NF-κB p65 (Ser536) (93H1) Rabbit mAb, NF-κB p65 (D14E12) XP Rabbit mAb, Phospho-IκBα (Ser32) (14D4) Rabbit mAb, IκBα (L35A5) Mouse mAb, Phospho-IKKβ (Ser176/180) (16A6) Rabbit mAb, IKKβ (D30C6) Rabbit mAb and TLR4, β-actin were provided by Cell Signaling Technology (Beverly, MA, United States). MyD88 (A0980) Rabbit pAb and TRAF6 (A0973) Rabbit pAb were purchased from ABclonal Biotechnology Co., Ltd. (Cambridge, MA, United States).

### Animals Experiments

BALB/c mice (25–30 g) were purchased from Experimental Animal Center of Huazhong Agricultural University (Wuhan, China). All animals were maintained in animal rooms at 22°C in a 12-h light/dark cycle and received food and water *ad libitum*. All animal experiments were performed in accordance with guidelines provided by the Laboratory Animal Research Center of Hubei province, and approved by the Ethical Committee on Animal Research at Huazhong Agricultural University (HZAUMO-2015-12). Mice were randomly divided into two groups (*n* = 12): control group and LPS group. The method for creating the LPS-induced ALI model was described previously (Cai et al., 2012). Briefly, LPS was diluted to 10 mg/mL with phosphate-buffered saline (PBS). The mice were intratracheally administered with LPS at the dose of 10 mg/kg body mass, and the control group received equal amount of PBS. After 24 h, the mice were euthanized with sodium pentobarbital, three mice in each group were selected to measure the wet and dry weight of the lungs, three other mice were used to evaluate histological changes, and the remaining six mice were used to perform molecular biological analyses.

### Histological Analysis

For histological analysis, lung tissues were excised and fixed with 4% paraformaldehyde for 24 h. Sections (4 μm) of the lungs were embedded in paraffin, sliced, and then stained with hematoxylin and eosin (H&E).

### Lung Wet to Dry Weight (W/D) Ratio and MPO Assays

The severity of pulmonary oedema was measured by calculating the W/D ratio of lung tissues. The lungs were excised, rinsed briefly in PBS, and then weighed to obtain the “wet” weight. Subsequently, the lung tissues were dried at 80°C for 24 h to obtain the “dry” weight. The lung W/D ratio was measured by dividing the wet weight by the dry weight. To detect the MPO activity, tissue samples were homogenized with reaction buffer (*w/v*, 1/9). The MPO activity was determined using an MPO determination kit according to the manufacturer’s protocols.

### Cell Culture

The murine macrophage cell line RAW264.7 cells and the human embryonic kidney cell line HEK293T cells were purchased from American Type Culture Collection (ATCC, Manassas, VA, United States). RAW264.7 macrophages were cultured in DMEM (Invitrogen, Carlsbad, CA, United States) supplemented with 10% fetal bovine serum (FBS; Sigma, St. Louis, MO, United States), streptomycin (50 μg/mL), and penicillin (50 U/mL) at 37°C in a 5% CO_2_ incubator. HEK293T cells were grown in DMEM supplemented with 10% FBS.

### CCK-8 Assay

The viability of macrophages after treatment with LPS (2 μg/mL) was assessed using a Cell Counting Kit-8 (CCK-8) assay kit purchased from Beyotime (Shanghai, China). In brief, macrophages were plated at a density of 4 × 10^4^ cells/well in 96-well plates for 1 h, and then the cells were treated with or without LPS for 0, 6, or 12 h. After treatment, 10 μL of CCK8 solution was added to each well and continued incubate for another 3 h. Finally, the optical density (OD) was measured at 450 nm on a microplate reader (Bio-Rad Instruments, Hercules, CA, United States).

### Cell Transfection

Cultured cells were seeded at 5 × 10^5^ cells/well in 6-well plates (Corning Inc., Corning, NY, United States) for 12 h before transfection. The cells were transfected with miR-181a mimics, miR-181a inhibitors, siRNA or the negative controls using Lipofectamine 2000 (Invitrogen, Carlsbad, CA, United States) according to the manufacturer’s instructions. The mimics, inhibitors, siRNA and corresponding negative controls were all synthesized by GenePharma (Shanghai, China). These sequences are shown in **Table 1**. The transfection efficiency was evaluated by qPCR assay.

**Table 1:** Sequences for miR-181a and TLR4 siRNA.

miR-181a mimics	Sense	5′-AACAUUCAACGCUGUCGGUGAGU-3′
	Antisense	5′-UCACCGACAGCGUUGAAUGUUUU-3′
Mimics NC	Sense	5′-UUCUCCGAACGUGUCACGUTT-3′
	Antisense	5′-ACGUGACACGUUCGGAGAATT-3′
miR-181a inhibitors	Sense	5′-ACUCACCGACAGCGUUGAAUGUU-3′
Inhibitors NC	Sense	5′-CAGUACUUUUGUGUAGUACAA-3′
TLR4 siRNA	Sense	5′-GGACAGCUUAUAACCUUAATT-3′
	Antisense	5′-UUAAGGUUAUAAGCUGUCCTT-3′
siRNA NC	Sense	5′-UUCUCCGAACGUGUCACGUTT-3′
	Antisense	5′-ACGUGACACGUUCGGAGAATT-3′

### ELISA

24 h after transfection with miR-181a mimics or inhibitors or the respective controls, the cells were then stimulated with 2 μg/mL LPS for 12 h. The levels of TNF-α, IL-1β, and IL-6 in the cell culture supernatants were measured with ELISA kits according to the manufacturer’s protocols.

### RNA Isolation and qPCR Analysis

Total RNA was isolated using the TRIzol reagent (Invitrogen, United States) in accordance with the manufacturer’s instructions. The concentration and purity of isolated RNA were evaluated by OD values at 260 and 280 nm using Q5000 (Quawell Technology, United States). The ratios of OD260 to OD280 for the samples were between 1.9 and 2.0. Typically, the ratio of OD260 to OD280 should range between 1.9 and 2.0 for good-quality RNA. Then all RNA samples were treated with RNase-free DNase to remove contaminating genomic DNA. For miRNA analysis, cDNA was synthesized by M-MLV reverse transcriptase with a special reverse transcription primer for miRNAs. PCR was performed using a miRNAs real-time PCR kit (GenePharma, Shanghai, China) according to the instructions of the manufacturer. The primers for miR-181a and U6 snRNA were purchased from GenePharma. For mRNA analysis, cDNA was synthesized using a PrimeScript RT reagent kit (Takara, Dalian, China) according to the manufacturer’s instructions. PCR was carried out using SYBR Green plus reagent kit (Roche, Basel, Switzerland) according to the instructions of the manufacturer. The sequences of the primers for PCR are as follows: TLR4, sense-TTCAGAGCCGTTGGTGTATC, antisense-CTCCCATTCCAGGTAGGTGT; IL-1β, sense-CCTGGGCTGTCCTGATGAGAG, antisense-TCCACGGGAAAGACACAGGTA; IL-6, sense-GGCGGATCGGATGTTGTGAT, antisense-GGACCCCAGACAATCGGTTG; TNF-α, sense-CTTCTCATTCCTGCTTGTG, anti-sense-ACTTGGTGGTTTGCTACG; GAPDH, sense-CAATGTGTCCGTCGTGGATCT; antisense-GTCCTCAGTGTAGCCCAAGATG. The relative expression levels of miR-181a and mRNAs were normalized to the endogenous references U6 snRNA and GAPDH following the 2^-ΔΔCt^ method.

### Western Blot Analysis

Total protein was extracted with RIPA reagent (BioSharp, China) according to the manufacturer’s recommended protocol. The total protein concentrations were determined using the Pierce BCA Protein Assay Kit (Thermo Fisher Scientific, Rockford, IL, United States). Protein samples (40 μg) were separated by 10% SDS–polyacrylamide gel electrophoresis (SDS–PAGE), transferred onto polyvinylidene difluoride (PVDF) membranes, and probed with primary antibodies against the indicated proteins (1:1000) overnight. Membranes were then washed and exposed to horseradish peroxidase-conjugated secondary antibodies (1:4000), and visualized using enhanced chemiluminescence.

### Luciferase Reporter Assay

To study the effect of miR-181a on the LPS-induced activation of NF-κB, a NF-κB luciferase reporter gene assay was carried out as described previously (Du et al., 2011). Briefly, macrophages were co-transfected with pNF-κB-Luc, pRL-TK control vectors and miR-181a mimics along with indicated controls. After 24 h, the cells were stimulated with 2 μg/mL LPS for another 12 h. The cells were lysed and analyzed for luciferase activities using the Dual-Luciferase Reporter Assay System (Promega, Madison, WI, United States) following the manufacturer’s protocols.

The possible sites of binding between TLR4 and miR-181a were predicted using TargetScan^1^ and miRanda^2^. To determine whether TLR4 is a direct target of miR-181a, we cloned 3′-UTR of TLR4 into a psiCHECK^TM^-2 vector (Promega, Madison, WI, United States) to generate a wild- or mutant-type TLR4 3′-UTR luciferase reporter vector. For the luciferase assay, HEK293T cells were co-transfected with the luciferase reporter vectors and miR-181a mimics or controls, respectively. After 24 h of transfection, the luciferase activities were measured.

### Reactive Oxygen Species (ROS) Assay

Intracellular ROS levels were detected using the fluorescent probe DCFH-DA (Beyotime, Shanghai, China). At the end of the treatment, cells were incubated with 10 μM DCFH-DA at 37°C for 30 min in the dark. The fluorescence intensity were then determined using an inverted fluorescence microscope (BX51TF, Olympus, Tokyo, Japan) or a flow cytometry (FACSCalibur, BD Biosciences, San Jose, CA, United States). For the fluorescence images, the integrated optical density (IOD) and area of cells were measured by Image-Pro Plus (IPP) 6.0 software (Media Cybernetics, Silver Spring, MD, United States), and the ROS fluorescence intensity was expressed as IOD/area. For the flow cytometry, the intracellular mean fluorescence intensity was analyzed for more than 10000 cells of each sample by FlowJo software (Tree Star, San Carlos, CA, United States).

### Immunofluorescence Staining

Lung tissues were fixed in 4% paraformaldehyde for 24 h and then embedded in paraffin. Tissue samples were permeabilized with PBS containing 0.3% Triton X-100 and 10% BSA. RAW264.7 macrophages (1 × 10^5^ cells/mL) were seeded onto a 12-well plates. After the cells were treated as indicated, immunofluorescence staining was performed. Sections of tissues or cells were incubated with special primary antibodies (1:100) overnight at 4°C and then incubated with FITC-labeled secondary antibodies (1:200) in the dark for 1 h at 25°C. Nuclei were stained using DAPI for 10 min, and fluorescent images were captured with an inverted fluorescence microscope. The IOD and area of cells were measured by IPP 6.0 software, and the fluorescence intensity was expressed as IOD/area.

### Statistical Analysis

Data are expressed as mean ± SEM. Statistical analysis was performed using the Student’s *t*-test, with values of *p* < 0.05 considered statistically significant.

## Results

### The Expression of miR-181a Is Reduced in the Lung Tissues of LPS-Challenged Mice

**FIGURE 1:**
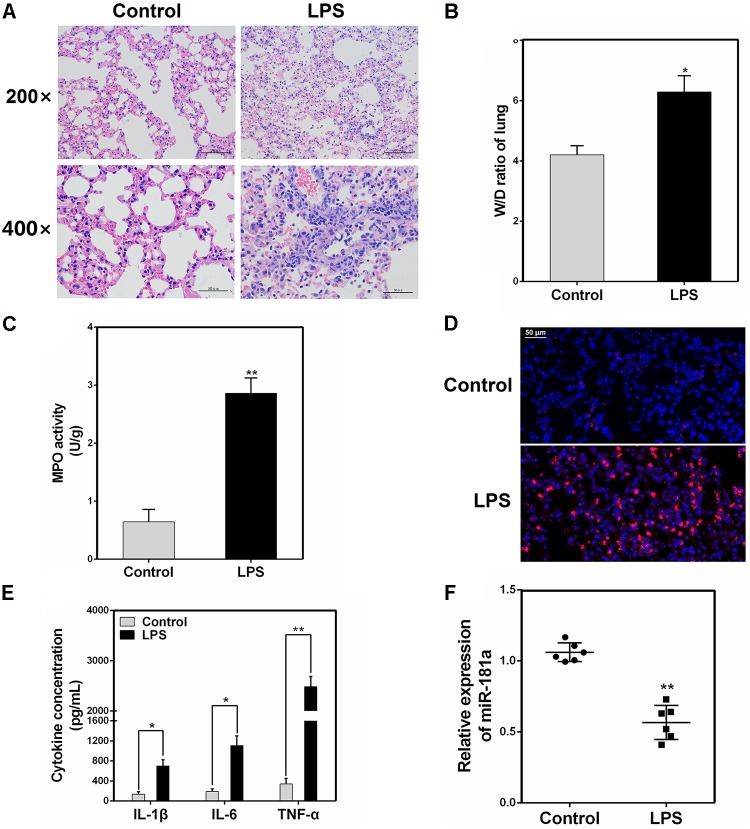
miR-181a is down-regulated in the lung tissues of LPS-induced ALI mice. **(A)** Histopathological analysis of lung tissues. Mice were intratracheally administered with LPS for 24 h, and the degree of inflammation of lung samples was assessed with H&E staining (*n* = 3). **(B)** Lung W/D ratio (*n* = 3). **(C,D)** Infiltration of neutrophils into the lung tissues was measured by myeloperoxidase (MPO) immunofluorescence staining and MPO activity (*n* = 3). **(E)** The levels of cytokines IL-1β, IL-6, and TNF-α was detected by ELISA (*n* = 3). **(F)** The miR-181a expression was detected in the lung tissues of LPS treated mice by qPCR (*n* = 6). U6 snRNA was used as an endogenous control. Data are expressed as mean ± SEM of three independent experiments. ^∗^*P* < 0.05; ^∗∗^*P* < 0.01 (Student’s *t*-test).

We induced ALI in mice with LPS and then performed H&E staining. As shown in **Figures 1A,B**, the control group displayed the normal pulmonary histology. In contrast, the lung tissues from the LPS group showed remarkable lung injury, including hemorrhage, interstitial edema and infiltration of inflammatory cells. These results were also further confirmed by subsequent MPO and ELISA results (**Figures 1C–E**). Some miRNAs play a pivotal role in regulation of inflammation response triggered by LPS (Biswas and Lopez-Collazo, 2009). In order to explore whether miR-181a is involved in this immune reaction, we measured the expression of miR-181a in the lung tissues of ALI mice. qPCR assay showed that the level of miR-181a was markedly suppressed in lung tissues of ALI mice when compared to that of the control group (**Figure 1F**).

### miR-181a Is Down-regulated in LPS-Stimulated Macrophages

**FIGURE 2:**
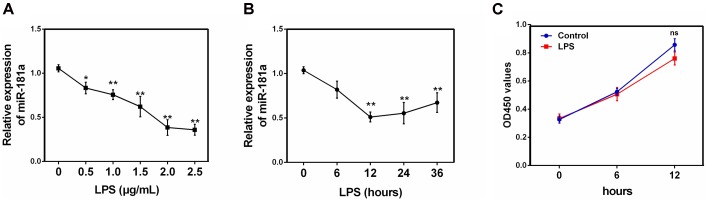
miR-181a is down-regulated in LPS-stimulated RAW264.7 macrophages. **(A)** Macrophages were stimulated with different concentrations of LPS for 12 h. **(B)** Macrophages were stimulated with 2 μg/mL LPS at different times as indicated. Cells were harvested, and miR-181a expression was measured by qPCR. The relative expression of miR-181a was normalized to U6 snRNA. **(C)** The viability of macrophages after treatment with LPS (2 μg/mL) was assessed using a CCK-8 assay kit. Data are expressed as mean ± SEM of three independent experiments. ^∗^*P* < 0.05; ^∗∗^*P* < 0.01 (Student’s *t*-test).

To investigate the effect of miR-181a in LPS-induced inflammation in ALI, the expression of miR-181a in LPS-stimulated RAW 264.7 macrophages was also detected. As displayed in **Figure 2A**, miR-181a expression was significantly decreased upon LPS stimulation and the down-regulation of miR-181a expression was dose-dependent. Besides, we also tested miR-181a expression at different time points of LPS treatment. Results showed that LPS decreased miR-181a expression in a time-dependent manner and reached a nadir at 12 h (**Figure 2B**). In addition, CCK-8 assay demonstrated that the cell viabilities were not affected by LPS at the concentration (2 μg/mL) used (**Figure 2C**). These results further confirm that miR-181a is involved in LPS-mediated immune response.

### miR-181a Decreases the LPS-Induced Production of Pro-inflammatory Cytokines

**FIGURE 3:**
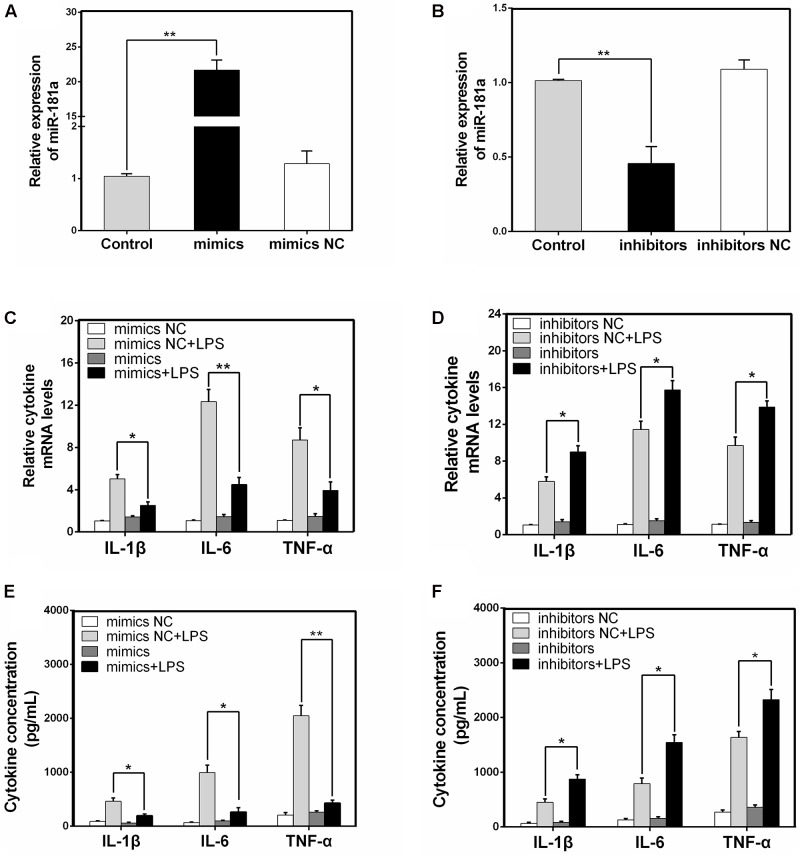
miR-181a decreases the LPS-induced production of pro-inflammatory cytokines. **(A,B)** Macrophages were transfected with miR-181a mimics or inhibitors. At 24 h post-transfection, miR-181a levels were measured by qPCR. The relative expression of miR-181a was normalized to U6 snRNA. **(C,D)** Cells were transfected with 50 nM miR-181a mimics or 100 nM miR-181a inhibitors for 24 h, and then stimulated with 2 μg/mL LPS for 12 h. The expression of cytokines IL-1β, IL-6, and TNF-α was determined by qPCR. GAPDH was used as an endogenous control. **(E,F)** The levels of cytokines IL-1β, IL-6, and TNF-α was detected by ELISA. Data are expressed as mean ± SEM of three independent experiments. ^∗^*P* < 0.05; ^∗∗^*P* < 0.01 (Student’s *t*-test).

It is well-known that LPS could activate NF-κB pathway and subsequently lead to the secretion of inflammatory cytokines such as IL-1β, IL-6, and TNF-α, which all promote the development of ALI (Wu et al., 2017). To unravel the specific role of miR-181a in cytokines production in LPS-induced inflammatory response, macrophages were transiently transfected with miR-181a mimics or inhibitors. Twenty-four hours after transfection, the transfection efficacy was assessed by qPCR, and the results confirmed that transfection with miR-181a mimics or inhibitors led to a dramatical increase or decrease in miR-181a expression (**Figures 3A,B**). Subsequently, the cells were stimulated with 2 μg/mL LPS for another 12 h and the levels of inflammatory cytokines were detected by qPCR and ELISA. As shown in **Figures 3C–F**, over-expression or inhibition of miR-181a significantly decreased or increased the LPS-induced secretion of pro-inflammatory cytokines. These results indicate that miR-181a play an anti-inflammatory role in the LPS-induced inflammatory response.

### miR-181a Suppressed LPS-Induced Activation of NF-κB Pathway

**FIGURE 4:**
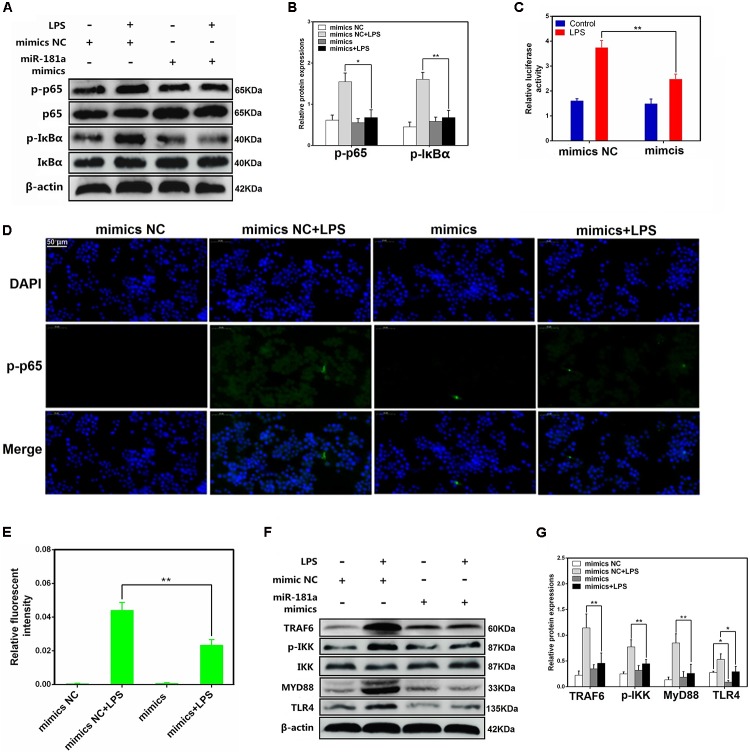
miR-181a suppressed LPS-induced activation of NF-κB pathway. **(A)** Macrophages were transfected with miR-181a mimics or mimics NC for 24 h, then stimulated with 2 μg/mL LPS for 12 h. **(B)** The protein levels of NF-κB p65 and IκBα were measured by western blotting. β-actin was used as an internal control. **(C)** The NF-κB luciferase activity was measured by dual-luciferase assay. **(D)** Translocation of the p65 subunit from the cytoplasm into the nucleus was assessed by immunofluorescence staining (×400), scale bar = 50 μm. Blue spots represent cell nuclei, and green spots indicate p-p65 staining. **(E)** The IOD and area of cells were measured by IPP 6.0 software, and the fluorescence intensity of p-p65 was expressed as IOD/area. **(F)** Cells were treated as **(A)**, and the protein levels of upstream molecules of NF-κB pathway were measured by western blotting. **(B,G)** Gray values of the indicated proteins were measured by Image-Pro Plus (IPP) 6.0 software. Data are expressed as mean ± SEM of three independent experiments. ^∗^*P* < 0.05; ^∗∗^*P* < 0.01 (Student’s *t*-test).

It has been generally accepted that the expression levels of inflammatory cytokines are regulated by multiple signaling pathways, such as NF-κB pathway. NF-κB, a crucial nuclear transcription factor, plays an important role in LPS-induced ALI (Jiang et al., 2017b). To further vindicate the mechanism of miR-181a in cytokines inhibition, we then determined the ability of miR-181a to modulate the activation of NF-κB pathway in macrophages. The protein levels of NF-κB p65 and IκBα were detected using western blotting. As shown in **Figures 4A,B**, the phosphorylated p65 and IκBα proteins were significantly increased in the LPS group. In contrast, their levels were dramatically reduced while over-expression of miR-181a. We also studied the effect of miR-181a in NF-κB activation by dual-luciferase assay, and the results showed that miR-181a inhibited NF-κB activation (**Figure 4C**). Similarly, immunofluorescence results confirmed that miR-181a mimics induced a decrease in the nuclear translocation of NF-κB p65 after 12 h of LPS stimulation when compared to the control mimics (**Figures 4D,E**). To find out the possible target of miR-181a, we next detected the expression of upstream molecules of NF-κB pathway. Western blot results showed that LPS-induced TLR4, MyD88, TRAF6 and phosphorylated IKKα/β levels were markedly inhibited. However, the TLR4 expression was also decreased by miR-181a mimics in the absence of LPS compared with the control mimics (**Figures 4F,G**). These data suggests that miR-181a could suppress LPS-induced activation of NF-κB pathway, likely through decreasing TLR4 expression.

### TLR4 Is a Molecular Target of miR-181a

**FIGURE 5:**
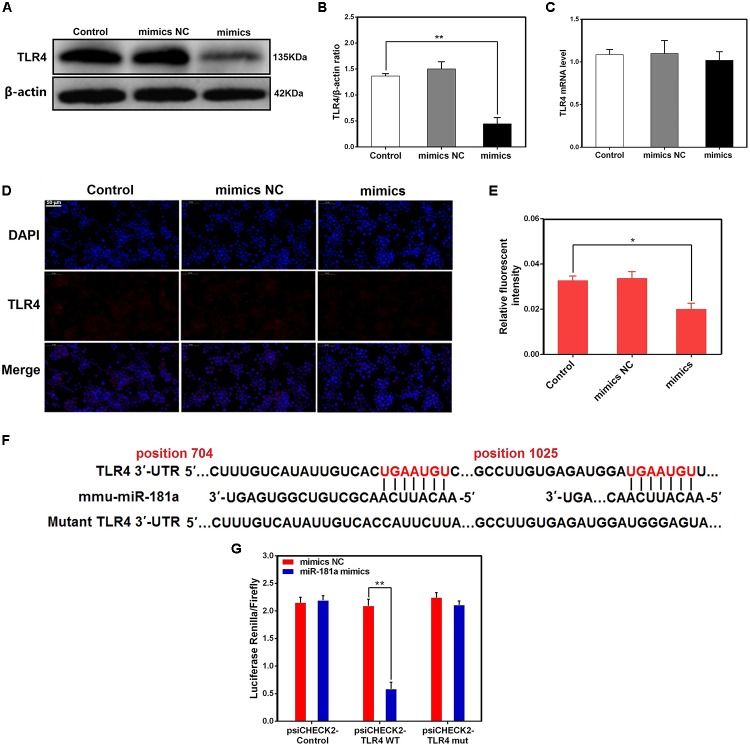
TLR4 is a molecular target of miR-181a. **(A)** Macrophages were transfected with miR-181a mimics or mimics NC for 48 h, and the protein level of TLR4 was measured by western blotting. β-actin was used as an internal control. **(B)** Gray values of TLR4 protein were measured by IPP software. **(C)** Cells were treated as **(A)**, the mRNA level of TLR4 was detected by qPCR. GAPDH was used as an internal control. **(D)** Immunofluorescence staining was performed to identify the expression of TLR4 (×400), scale bar = 50 μm. Blue spots represent cell nuclei, and red spots indicate TLR4 staining. **(E)** The fluorescence intensity of TLR4. **(F)** The alignment of miR-181a and TLR4 3′-UTR by computational prediction via the TargetScan and miRanda. **(G)** The dual-luciferase reporter assay was performed in 293T cells. Cells were co-transfected with the wild- or mutant-type TLR4 3′-UTR luciferase reporter vectors, as well as miR-181a mimics or mimics NC. The ratio of Renilla activity/Firefly activity represents luciferase activity. Data are expressed as mean ± SEM of three independent experiments. ^∗^*P* < 0.05; ^∗∗^*P* < 0.01 (Student’s *t*-test).

We transfected macrophages with miR-181a mimics or negative controls and then examined the expression of TLR4. As displayed in **Figures 5A–E**, transfection with miR-181a mimics restrained the protein level of TLR4 but not the mRNA level, indicating that miR-181a may function at the translational level. In addition, bioinformatic softwares (TargetScan and miRanda) showed that there are two putative binding sites between miR-181a and the 3′-UTR of TLR4 (**Figure 5F**). To further confirm that miR-181a is able to directly bind to TLR4 mRNA, a luciferase reporter assay was performed. Briefly, the wild- or mutant-type TLR4 3′-UTR luciferase reporter vectors were transfected into HEK293T cells, and then treated with miR-181a mimics or control mimics. We found that miR-181a mimics markedly decreased the luciferase activity for the wild-type 3-UTR of TLR4 but showed no inhibition effect for the mutated 3′-UTR of TLR4 (**Figure 5G**). These results imply that miR-181a inhibits TLR4 expression by directly binding the 3′-UTR of TLR4 mRNA.

### Knockdown of TLR4 Alleviates LPS-Induced Inflammatory Responses

**FIGURE 6:**
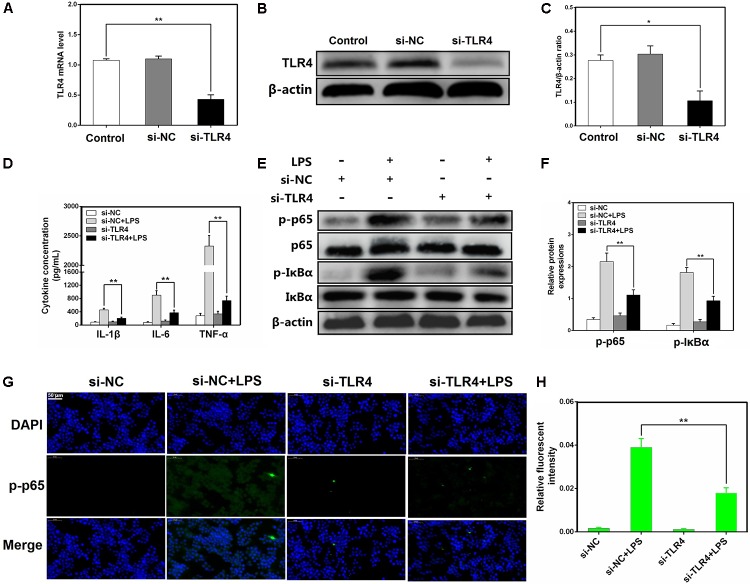
Knockdown of TLR4 alleviates LPS-induced inflammatory responses. **(A)** Macrophages were transfected with the siRNA specific for TLR4 (si-TLR4) or the negative control siRNA (si-NC) at a concentration of 200 nM for 24 or 48 h, and the mRNA level of TLR4 was measured by qPCR. GAPDH was used as an internal control. **(B)** The protein level of TLR4 was determined by western blotting. β-actin was used as an internal control. **(D)** Cells were transfected with 200 nM si-TLR4 or si-NC for 24 h, and then stimulated with 2 μg/mL LPS for 12 h. The levels of cytokines IL-1β, IL-6, and TNF-α were detected by ELISA. **(E)** The protein levels of NF-κB p65 and IκBα were measured by western blotting. β-actin was used as an internal control. **(G)** Translocation of the p65 subunit from the cytoplasm into the nucleus was assessed by immunofluorescence staining (×400), scale bar = 50 μm. Blue spots represent cell nuclei, and green spots indicate p-p65 staining. **(H)** The fluorescence intensity of p-p65. **(C,F)** Gray values of the indicated proteins were measured by IPP 6.0 software. Data are expressed as mean ± SEM of three independent experiments. ^∗^*P* < 0.05; ^∗∗^*P* < 0.01 (Student’s *t*-test).

TLR4 has been shown to play an essential role in LPS-mediated NF-κB activation (Guijarromuñoz et al., 2014; Jiang et al., 2017a). To further elucidate the mechanisms by which miR-181a regulates LPS-induced inflammatory responses, a siRNA specific for TLR4 (si-TLR4) was used to knock down TLR4 expression in macrophages, and then, the expression level of TLR4 was measured by qPCR and western blotting. Following transfection, TLR4 mRNA and the protein levels were significantly reduced (**Figures 6A–C**). Moreover, the production of the pro-inflammatory cytokines including IL-1β, IL-6, and TNF-α was also repressed (**Figure 6D**). Knockdown of TLR4 also significantly inhibited the phosphorylation of NF-κB p65 and IκBα, consistent with the above results (**Figures 6E–H**). Taken together, these findings strongly demonstrate miR-181a is implicated in the negative regulation of LPS-induced inflammation through inhibiting TLR4-NF-κB pathway.

### miR-181a Reduces LPS-Induced Intracellular ROS Accumulation in Macrophages

**FIGURE 7:**
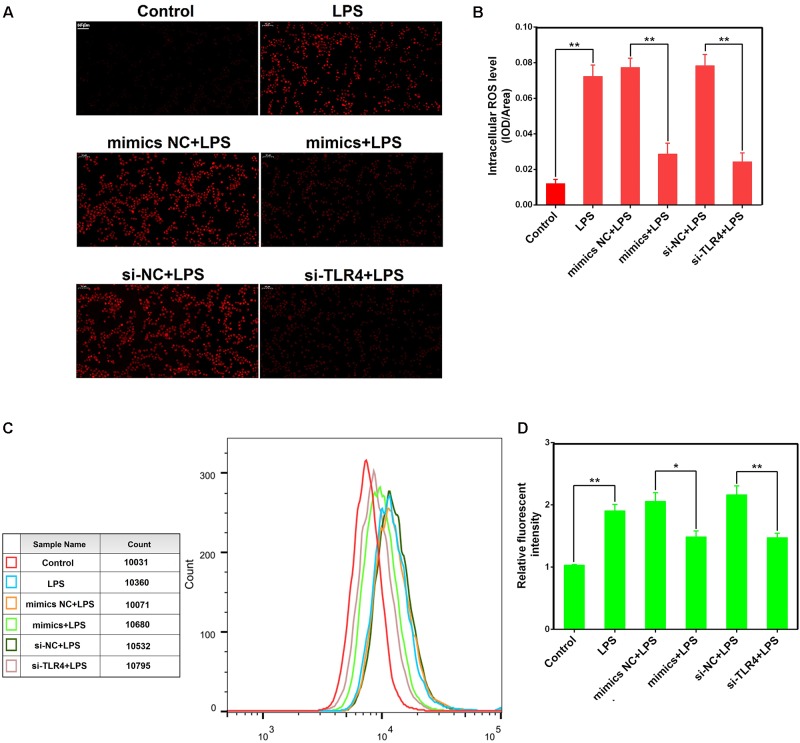
miR-181a reduces LPS-induced intracellular ROS accumulation in macrophages. Macrophages were transfected with miR-181a mimics or si-TLR4 or the respective controls for 24 h, then incubated with 10 μM DCFH-DA for 30 min followed by stimulation with 2 μg/mL LPS for an additional 30 min. **(A)** Qualitative characterization of ROS were viewed using an inverted fluorescence microscope. **(B)** The IOD and area of cells were measured by IPP 6.0 software, and the ROS fluorescence intensity was expressed as IOD/area. **(C)** The ROS levels of cells were detected using flow cytometry. **(D)** The relative fluorescence intensity was analyzed by FlowJo software. Data are expressed as mean ± SEM of three independent experiments. ^∗^*P* < 0.05; ^∗∗^*P* < 0.01 (Student’s *t*-test).

**FIGURE 8:**
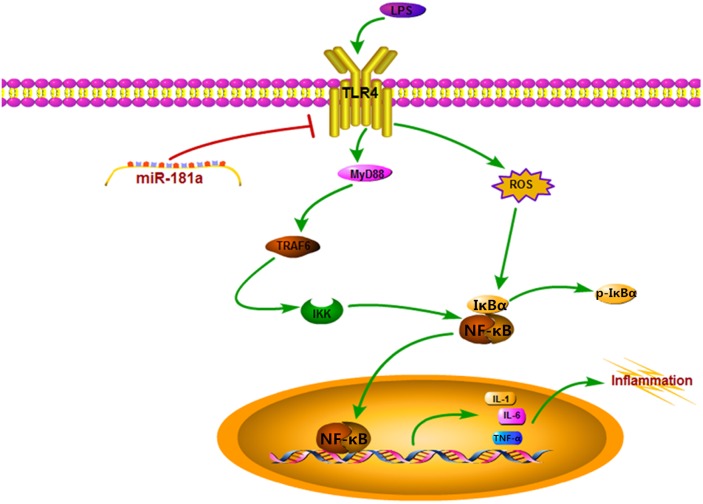
Schematic diagram of signaling pathways related to anti-inflammatory effects of miR-181a on LPS-induced inflammation.

Reactive oxygen species were involved in LPS-mediated immune response, and contributes to the exacerbation of inflammation (Zou et al., 2013). We observed that miR-181a mimics significantly restrained the accumulation of intracellular ROS in LPS-stimulated macrophages compared with mimics NC. Similarly, si-TLR4 also significantly decreased ROS production compared to si-NC (**Figures 7A–D**). These results reveal that miR-181a could also alleviate the inflammatory response mediated by inhibition of the ROS production (**Figure 8**).

## Discussion

Acute lung injury is a type of severe inflammatory disease, which is hard to treat and poor prognosis (Matthay and Zimmerman, 2005). Although multiple promising pharmacological interventions have been studied in patients with ALI, the mortality rate is still as high as 30–70% (Rubenfeld et al., 2005). Therefore, there is a need to develop a new therapeutic strategy for the treatment of ALI. miRNAs are an important class of small endogenous non-coding RNAs that play a regulatory role in various basic biological processes, such as cell proliferation, differentiation, apoptosis, and inflammation (Cheng et al., 2005; Ochs et al., 2011). It was found recently that some miRNAs can serve as a negative feedback regulator of inflammatory response, through targeting signaling proteins involved in signal transmission (Case et al., 2011; Iyer et al., 2012).

miR-181a belongs to the miR-181 family, and its nucleic acid sequence is highly conserved in mammals (Ji et al., 2009). Previous research has shown that miR-181a is mainly involved in modulation of tumor cell growth, and acts as a tumor suppressor in human glioma cells (Shi et al., 2008). More notably, anti-inflammatory function of miR-181a has been identified in some recent studies. miR-181a suppresses TNF-α-induced transcription of pro-inflammatory genes in liver epithelial cells by targeting p300/CBP-associated factor (Zhao et al., 2012), and inhibits oxidized low-density lipoprotein-stimulated inflammatory responses in dendritic cells by targeting c-Fos (Wu et al., 2012). Moreover, the immunoregulatory effect of miR-181a in differentiation of T helper cell and activation of macrophages has also been proposed (Ghorbani et al., 2017). However, we are not certain whether miR-181a has an anti-inflammatory effect in LPS-induced inflammation in ALI. Therefore, we employed an *in vivo* ALI model as well as an *in vitro* inflammation model using RAW 264.7 macrophages to explore the possible role of miR-181a in inflammatory response induced by LPS.In the current study, we found that exposure to LPS led to severe pathological lesions, including alveolar damage and inflammatory cell infiltration. Subsequently, we demonstrated that miR-181a is down-regulated in the lung tissues of LPS-challenged mice, consistent with a previous study (Cai et al., 2012). However, this finding is contrary to another published report which has suggested that miR-181a is up-regulated in the lung tissues (Li et al., 2016). A possible explanation for this might be that we used a lower dose of LPS (10 mg/kg) in the present study. Furthermore, down-regulation of miR-181a in macrophages stimulated with LPS in a dose- and time-dependent manner was also found, indicating miR-181a may have an important biological function in LPS-induced inflammation.

It is well recognized that innate immune system can recognize conserved pathogen associated molecular patterns (PAMPs) through pattern recognition receptors (PRRs), and is the main contributor to inflammation caused by pathogenic microbial infection (Akira and Hemmi, 2003; Zhou et al., 2014). TLR4 is one of the best studied PRR families expressed in macrophages and other immune cells, and evidence suggests that several PAMPs such as LPS can stimulate TLR4, which ultimately leads to the secretion of inflammatory mediators from macrophages (Shi et al., 2006; Lu et al., 2008). In the present study, we identified that over-expression or inhibition of miR-181a significantly decreased or increased the LPS-induced production of IL-1β, IL-6, and TNF-α in macrophages. NF-κB is a type of critical nuclear transcriptional regulatory factor responsible for the production of pro-inflammatory cytokines, which induce a cascade of inflammatory responses and related lung damage (Lawrence, 2009; Shen et al., 2009). Intriguingly, the activation of NF-κB pathway by LPS was also repressed following transfection with miR-181a mimics. The observations showed that miR-181a likely provided a negative feedback to inflammation stimulated by LPS.

In order to explore the programmed feedback mechanism of miR-181a regulating inflammation, it is essential to study its target genes. TLR4 plays an essential role for the LPS triggered-NF-κB activation (Guijarromuñoz et al., 2014). Knock-out of TLR4 could attenuate the pro-inflammatory state of diabetes in mice (Devaraj et al., 2011). More importantly, TLR4 possesses a central role in initiating changes in miRNAs expression in answer to invading pathogens (Curtale et al., 2013). Therefore understanding the regulatory effect of miRNA in TLR4 gene expression is vital for future development of therapeutic agents against inflammatory diseases. In present study, we found that miR-181a mimics dramatically decreased the TLR4 protein level. Furthermore, bioinformatics predictions made with TargetScan and miRanda showed that TLR4 is a putative target of miR-181a. To further validate that TLR4 is a molecular target of miR-181a, the luciferase reporter assay was performed. Luciferase activity was significantly reduced when co-transfected miR-181a mimics with wild-type TLR4 3′-UTR vector, whereas no significant change was observed with mutant-type TLR4 3′-UTR vector. These results imply that miR-181a is able to bind to TLR4 mRNA directly and inhibits its translation. Moreover, TLR4 expression was also silenced using si-TLR4 so as to further verify whether TLR4 is involved in the anti-inflammatory effect of miR-181a. Our results showed that knock-down of TLR4 ameliorated inflammatory response and NF-κB p65 phosphorylation under LPS stimulation. Collectively, these data strongly demonstrate that the TLR4 was the very target of miR-181a following LPS stimulation.

It is well-known that inflammation induced by endotoxin such as LPS was closely associated with ROS generation (Patruno et al., 2015). ROS, which are mainly produced by NADPH oxidase, are commonly considered cytotoxic and can even induce cell damage in high levels (Ray et al., 2012). Previous studies also have shown that ROS were implicated in TLR4-mediated immune reactions (Zhang et al., 2016; Pan et al., 2017), and elicited a cascade of inflammatory events, such as activation of NF-κB (Yu et al., 2015; Singh et al., 2016). In the present study, miR-181a mimics markedly suppressed the intracellular ROS accumulation in LPS-induced macrophages. As expected, si-TLR4 also decreased ROS production. These results suggest that miR-181a could also alleviate LPS-induced inflammation through reducing TLR4-mediated ROS production.

## Conclusion

The results of our study not only demonstrates that miR-181a targets TLR4 directly to regulate the activation of NF-κB and subsequent secretion of inflammatory cytokines in response to LPS stimulation, but also reveal further that this miRNA represses the intracellular ROS accumulation. All these findings indicates, that miR-181a may be a negative regulator of LPS-stimulated inflammation via the suppression of ROS generation and TLR4-NF-κB pathway.

## Author Contributions

KJ and GZ conceived and designed the experiments. TZ and YY carried out the experiments. SG, HW, and AS analyzed the data. KJ and GD wrote the manuscript. All authors agreed to be responsible for the content of the work.

## Conflict of Interest Statement

The authors declare that the research was conducted in the absence of any commercial or financial relationships that could be construed as a potential conflict of interest.

## Footnotes

1 www.targetscan.org

2 www.microrna.org
